# The Rey Auditory Verbal Learning Test: Cross-validation of Mayo Normative Studies (MNS) demographically corrected norms with confidence interval estimates

**DOI:** 10.1017/S1355617722000248

**Published:** 2022-04-28

**Authors:** David W. Loring, Jessica L. Saurman, Samantha E. John, Stephen C. Bowden, James J. Lah, Felicia C. Goldstein

**Affiliations:** 1 Department of Neurology, Emory University, School of Medicine, Atlanta, USA; 2 Department of Pediatrics, Emory University, School of Medicine, Atlanta, USA; 3 Department of Brain Health, University of Nevada, Las Vegas, USA; 4 Melbourne School of Psychological Sciences, University of Melbourne, Australia

**Keywords:** leaning and memory, confidence intervals, reliability and validity, demographic correction, predicted true score

## Abstract

**Objective:**

The Mayo Normative Studies (MNS) represents a robust dataset that provides demographically corrected norms for the Rey Auditory Verbal Learning Test. We report MNS application to an independent cohort to evaluate whether MNS norms accurately adjust for age, sex, and education differences in subjects from a different geographic region of the country. As secondary goals, we examined item-level patterns, recognition benefit compared to delayed free recall, and derived Auditory Verbal Learning Test (AVLT) confidence intervals (CIs) to facilitate clinical performance characterization.

**Method:**

Participants from the Emory Healthy Brain Study (463 women, 200 men) who were administered the AVLT were analyzed to demonstrate expected demographic group differences. AVLT scores were transformed using MNS normative correction to characterize the success of MNS demographic adjustment.

**Results:**

Expected demographic effects were observed across all primary raw AVLT scores. Depending on sample size, MNS normative adjustment either eliminated or minimized all observed statistically significant AVLT differences. Estimated CIs yielded broad CI ranges exceeding the standard deviation of each measure. The recognition performance benefit across age ranged from 2.7 words (*SD* = 2.3) in the 50–54-year-old group to 4.7 words (*SD* = 2.7) in the 70–75-year-old group.

**Conclusions:**

These findings demonstrate generalizability of MNS normative correction to an independent sample from a different geographic region, with demographic adjusted performance differences close to overall performance levels near the expected value of *T* = 50. A large recognition performance benefit is commonly observed in the normal aging process and by itself does not necessarily suggest a pathological retrieval deficit.

The Rey Auditory Verbal Learning Test (AVLT) (Rey, 1958; Taylor, 1959) is a common neuropsychological measure of verbal learning and memory and enjoys a long history of use that, despite its common eponym, has its origins in the late 19th century with the Swiss psychologist Édouard Claparède (Boake, 2000). Claparède developed the *Test de mémoire des mots* (Test of Memory for Words) as a single trial memory task containing 15 words. Claparède’s memory stimuli formed the basis of Rey’s multi-trial verbal learning test (Boake, 2000), although several words from Claparède’s/Rey’s list were modified in the translation from French to English (bell for belt, moon for sun, nose for moustache).

In North America, the AVLT is less frequently used than the California Verbal Learning Test (CVLT) in clinical settings to assess verbal learning and memory (Rabin et al., 2016), and there are clear psychometric and standardization advantages associated with the CVLT. Because the AVLT was developed as an instrument to research memory rather than created as a clinical memory test and remains in the public domain, the AVLT has never been subjected to contemporary standardization practices. Consequently, for clinicians using the AVLT in their practices and for research protocols using the AVLT for sample characterization, there are multiple datasets to choose from for normative characterization (Mitrushina et al., 2005). However, the normative sampling and subject description of these normative datasets do not meet the formal standards required from commercial test publishers such as standardization and characterization of validity, reliability, and errors of measurement.

Until recently, the two main sources for AVLT normative values were the Schmidt AVLT meta-norms (aggregate adult sample of nearly 2000 participants; Schmidt, 1996) and the Mayo Clinic’s Older Americans Normative Studies (MOANS; derived from 530 cognitively normal participants living in Olmstead County, Minnesota; Ivnik et al., 1992). In a major improvement for AVLT normative characterization, the Mayo Normative Studies (MNS) provides demographically characterized normative information from a large sample of 4400+ cognitively healthy participants living in the Rochester, Minnesota area (Stricker et al., 2021). The MNS cohort demonstrated, in addition to age and education effects, robust sex performance differences across multiple AVLT measures, highlighting the importance of demographic sex correction to accurately characterize AVLT performance. While group differences for sex are incorporated into CVLT normative tables, most existing AVLT norms have not characterized test performance by sex despite this being recognized as an important normative consideration (Gale et al., 2007). Thus, there are clear risks of different clinical inferences based upon the choice of normative datasets and incorporation of appropriate demographic corrections.

Another important factor influencing AVLT interpretation is the reliability of the obtained memory scores. Consideration of confidence intervals (CIs), however, is often neglected during test score interpretation. Nunnally and Bernstein (1994) note “it is important to recognize that any obtained score is only one in a probable range of scores whose size is inversely related to the test’s reliability” (p. 291). Lezak (1994) also observes that “few persons unschooled in statistics understand measurement error; they do not realize that two different numbers need not necessarily stand for different quantities but may be chance variations in the measurement of the same quantity” (p. 132). Consideration of CIs can influence whether specific diagnostic thresholds have been met, and score uncertainty has been incorporated into the Fifth edition of the Diagnostic and Statistical Manual of Mental Disorders (DSM-V; American Psychiatric Association, 2013) in which an error of 5 IQ points was explicitly included in defining the upper range of cognitive or intellectual disability to reflect measurement error.

We report AVLT performance from 663 cognitively healthy volunteers aged 50 years or older who were participants in the Emory Healthy Brain Study (EHBS; Goetz et al., 2019). AVLT data were analyzed to: (1) replicate the magnitude of sex differences reported in the MNS sample; and (2) establish the generalizability of MNS demographic normative correction to cognitively healthy participants from a major metropolitan southeastern city in the United States. Geographic region is one potential factor contributing to different clinical inferences from independent normative samples (Martin et al., 2017). As secondary goals, we (1) examine item-level patterns to explore whether specific words are disproportionate contributors to any age-, sex-, or education-related effects; (2) characterize performance levels for individual targets and foils during recognition memory testing; (3) examine the recognition performance benefit compared to delayed free recall across age groups; and (4) derive AVLT CIs from MNS reliability statistics to facilitate clinical performance characterization.

## Methods

### Participants

Participants were subjects in the EHBS and were tested between April 2016 and December 2020. The EHBS is designed as a preclinical Alzheimer disease (AD) biomarker discovery project intended to capture early conversion from normal age-related cognitive performance. The EHBS cohort is a large community-based prospectively enrolled cohort of cognitively healthy participants between 50–75 years of age (Goetz et al., 2019). Although the study protocol limited enrollment of subjects up to age 75 years, during the initial study ramp up, several subjects over age 75 were allowed to enroll (*n* = 5) and their scores are included in this report. Participants were self-declared cognitively normal without functional limitation, had normal Montreal Cognitive Assessment (MoCA) scores (Nasreddine et al., 2012), and were without neurological diagnoses suggesting prodromal or current degenerative disease. All patients spoke fluent English. This project was approved by the Emory University institutional review board in accordance with the Declaration of Helsinki and all participants provided written informed consent.

There were 663 participants with MoCA scores that were 24/30 or higher, and included 463 females and 200 males. Participants with MoCA scores less than 24/30 were excluded (*n* = 72). The average education level for females was 16.6 years (*SD* = 2.0) and for males was 16.9 years (*SD* = 2.0). The average age for females was 62.6 years (*SD* = 6.6) and 63.7 (*SD* = 6.9) for males. There were 20 participants who identified as Hispanic and 643 who identified as non-Hispanic. The largest group of participants identified as White (*n* = 584) followed by Black (*n* = 69), American Indian or Alaska Native (*n* = 3), Asian (*n* = 3), or Mixed (*n* = 1), with 3 participants choosing not to disclose. There were 400 White and 54 Black females and 184 White and 15 Black male participants.

### Auditory Verbal Learning Test

The AVLT is a verbal learning and memory task in which the individual is asked to learn a list of 15 semantically unrelated words (List A) over five learning trials. After the fifth trial, a new list of 15 words is presented for a single learning trial (List B), followed by free recall of the original 15 items (List A). Delayed free recall (~ 30 min) for the original List A items is obtained followed by a recognition trial. The recognition memory task was developed by Ivnik et al. (1992) (Schmidt Form AB), which itself is a modification of Rey’s paragraph recognition format presented by Lezak (1976). Thirty words consisting of the 15 List A targets and 15 foils are presented as a two-column list, and the participant indicates words considered to be from the List A stimulus set. The interval prior to AVLT delayed memory testing included Rey-Osterrieth Complex Figure, Digit Span, Trail Making Test, and Judgment of Line Orientation.

### Analysis

Group differences for age, sex, and education were established based upon one-way ANOVAs for each group separately for primary AVLT measures. We did not impose any experiment-wise alpha adjustment associated with multiple comparisons since in the context of the present report, we considered Type II errors more serious than Type I errors (Perneger, 1998). Effect sizes are reported using eta squared (*η*^*2*^); by convention, *η*^*2*^ ≥ .01 is considered a small effect, *η*^*2*^ ≥ .06 is considered a medium effect, and *η*^*2*^ ≥ .14 is considered a large effect.

Additional analyses were performed for recognition items including both target words and foils, with statistical demographic performance differences established using *chi*-squared analyses. The recognition performance benefit compared to delayed free recall was analyzed using age as the group factor with a one-way within subject ANOVA, with no correction for false positive intrusion errors.

### Confidence interval construction

Although AVLT reliabilities were reported for ~80% of the MNS subjects (*n* = 3,555), formal CIs were not reported (Strieker et al., 2021). Using the MNS test-retest reliabilities, we calculate CIs to facilitate clinical interpretation. We do not use the standard error of measurement as the basis for constructing CIs around test scores since it provides inaccurate estimates of the confidence or prediction intervals, especially with lower reliabilities (Dudeck, 1979; Nunnally & Bernstein, 1994). CIs are estimated for primary AVLT score using MNS test-retest Pearson reliabilities (Strieker et al., 2021, Table 3) and MNS raw score Standard Deviations (Stricker et al., 2021, Supplemental Table 1) to calculate *SE*_Estimation_ and *SE*_Prediction_ for raw scores and *T* scores, respectively. *SE*_Estimation_ is calculated using this formula [σ $\sqrt{r_{xx}\;(1-r_{xx})}$] and *SE*_Prediction_ is calculated using this formula [σ $\sqrt{1-(r_{xx}*r_{xx})}$], where σ is the standard deviation and *r*_*xx*_ is the reliability of the test score (Bowden & Finch, 2017). The CI is a matter of professional judgment, some clinicians preferring a 90% CI, others a 95% CI, and others some other value for the CI; here we use *z* = 1.64 for 90% CI generation.

## Results

### Primary analyses

#### Age effects

To characterize age-related influences on raw AVLT performance, participants were grouped into six 5-year age bands beginning with 50–54. One-way ANOVAs were performed on raw AVLT scores including Sum of Trials 1–5, 3-Trial Sum, List B Recall, Immediate List A Recall, Delayed List A Recall, and Recognition variables including Correct Targets, False Positives, and Recognition/Discrimination. While 3-Trial Sum, reflecting the trial sum across the initial 3 AVLT learning trials, is not a common AVLT score, the 3-Trial Sum is included in the MNS regression equations. Characterizing the 3-Trial Sum provides interpretative guidance for the 3-trial AVLT short-form, which is a supplemental NIH Cognitive Toolbox measure (National Institutes of Health & Northwestern University, 2017). Except for False Positive Recognition errors, there were significant age effects across all ALVT measures (Table 1). These findings confirm the well-established age-decline across multiple memory measures and provide reassurance regarding the representativeness of our EHBS sample. MNS normative performance adjusting for age-related changes is also presented in Table 1. In contrast to raw scores, no age-related differences were observed when comparing MNS demographically corrected *T* scores.

#### Sex differences

One-way ANOVAs with sex as the grouping factor were performed separately on AVLT measures including Sum of Trials 1–5, 3-Trial Sum, List B Recall, Immediate List A Recall, Delayed List A Recall, and Recognition including Correct Targets, False Positives, and Recognition/Discrimination (Targets minus False Positives). All AVLT scores showed statistically significant group sex differences at the *p* < .001 levels of statistical significance or better except for False Positives, which was statistically significant but with a lower probability level (*p* = .009). Effect sizes ranged from *η*^*2*^ = .01 (False Positive Recognition Errors) to *η*^*2*^ = .07 (Trial 1–5 Sum) (see Table 2).

We next investigated sample similarity to the MNS norms by calculating demographically corrected *T* scores for the primary AVLT measures separately for each sex. If the MNS normative sample is generalizable across geographic region, then demonstrated sex differences present with raw performance levels should no longer be observed, and average values for both men and women across all AVLT measures following transformation should approach *T* = 50 and a *SD* = 10. After full MNS demographic correction (age, sex, and education), most sex differences were no longer present, with the only remaining statistically significant sex differences being Trial 1–5 Sum (*p* = .036) and List B recall (*p* = .046) (see Table 2). Although Trial 1–5 and List B recall differences remain statistically significant, the statistical significance results from the relatively large sample sizes associated with small magnitude effects of *η*^*2*^ = 0.007 and *η*^*2*^ = 0.003, respectively.

#### Education differences

To characterize education-related influences on raw AVLT performance, participants were classified into groups (12 years, 13–15 years, 16–17 years, and 18+ years; see Table 3). Significant group differences were present for Trial 1–5 Sum (*p* = .004), 3-Trial Sum (*p* = .009), List B (*p* = .004), and False Positive Recognition Hits (*p* = .045). Application of MNS norms eliminated any education group performance differences for available measures (Trial 1–5 Sum [*p* = .901], 3-Trial Sum [*p* = .635], and List B [*p* = .441]).

### Secondary analyses

#### Item-level learning

**Figure 1.**
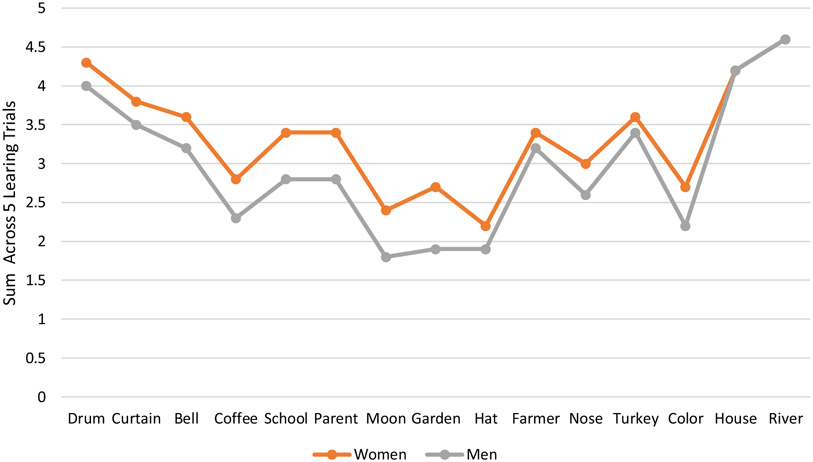
Individual item performance levels across learning trials by sex.

To investigate the source of sex differences on AVLT summary scores, we explored whether sex group differences were present at the individual word level by examining the 5-trial sums for each word individually. Statistically significant sex differences were present for all words except for farmer, house, and river, with the largest effect sizes present for garden (*η*^*2*^ = 0.06) and moon (*η*^*2*^ = 0.04, see Figure 1, Table 4).

We performed a similar series of ANOVAs for age group analyzing the 5-trial sums for each word individually. Statistically significant age effects were present for all words with the exception of *hat* and *river* ranging in level of statistical significance from *p* = .043 (*house*) to *p* = .0001 (*farmer*); effect sizes for all List A words are presented in Table 4.

#### Item-level recognition

Correct individual item recognition for targets ranged from 78.3% (*house*) to 98.5% (*farmer*) (Table 5). Incorrect identification of foils ranged from 0% (*kerchief*, *broomstick*) to 46.2% (face). The high frequency of incorrectly choosing *face* results from the MoCA being administered prior to the AVLT, where *face* is one of the 5 MoCA memory stimuli. The next most selected foil was *teacher* at 22.4% followed by *gun* (12.3%).

Age group differences for individual items using *chi*-squared analyses were present for *curtain* (*p* = .028) and *parent* (*p* = .023). There were no age group differences in foil identification or with other target words.

Sex differences for individual item recognition were examined using chi-squared analyses. Significant sex recognition effects included *teacher* (*p* = .04), *moon* (*p* = .0001), *color* (*p* = .01), *coffee* (*p* = .015), *hat* (*p* = .0001), *turkey* (*p* = .0002), *nose* (*p* = .003), *bell* (*p* = .003), *garden* (*p* = .002), and *parent* (*p* = .037). There were no significant sex differences for any foil.

Education differences were explored after combining the two groups with less than a college education into a single group due to small cell sizes associated in both high school and less than college education groups. For items with all cell sizes greater than 5 in each cell, group differences were present for *nose* (*p = .016*) and *face* (=.*044*), both of which were associated with more incorrect recognitions with the low education group.

#### Recognition memory benefit

Because the performance benefit of recognition testing compared to delayed free recall is frequently considered an indication of memory retrieval inefficiency, we examined the recognition benefit compared to delayed free recall (Recognition correct – Delay Free Recall) as a function of age. We made no correction for false positive (commission) errors; 92.7% of the sample made 2 or fewer intrusion errors. The sex x age group interaction was not statistically significant, and therefore we report performances with sexes combined. The performance benefit across age demonstrated a medium effect size (*η*^*2*^ = 0.066) ranging from 2.7 words (*SD* = 2.3) in the 50–54 age group to 4.7 words (*SD* = 2.7) in the 70–74-year-old age group. Performance for each age group is presented in Table 6.

#### Confidence intervals

MNS test-retest reliability coefficients derived from slightly over 80% of the full MNS normative sample with follow-up testing (*M* = 16.7 months, *R* = 8.1–37.3) were used to calculate AVLT CIs (Table 7). Also shown are CIs derived from the standard error of prediction (*SE*_*Prediction*_) for change scores associated with repeated testing. The respective CI is centered on the *predicted true score* during the initial assessment for both the single assessment and interval change score (see Bowden & Finch, 2017). Note that the prediction interval (or CI) derived from the standard error of prediction (*SE*_*Prediction*_) is a variant of the formula for predicting the range of scores at retest using “reliable change” methods (Hinton-Bayre & Kwapil, 2017). For both single assessment and characterization of follow-up change scores, the 90% CIs are large and typically exceed 1 *SD* for single scores and 2 *SDs* for interval change scores.

## Discussion

These findings confirm AVLT sex differences reported by Stricker et al. (2021) which, by extension, demonstrates how different clinical inferences may be made based solely on the normative database selected to characterize performance. Although AVLT sex differences have previously been described (Gale et al., 2007), common AVLT normative tables do not demographically correct for sex. The failure of other datasets to correct for sex provides prima facie evidence of risk of different clinical inference across various normative approaches even in the absence of direct formal statistical performance contrasts. However, Stricker et al. described 3.1% of females and 13.0% of males being characterized as having low test performance on 30-min recall using MOANS (*ss* < 7) with no differences when fully adjusted using MNS based upon *T* < 40 (female = 13.8%; male = 13.7%; Supplemental Table 4). Application of the full MNS demographic correction in the EHBS cohort adjusted for the sex differences across most measures, and the small statistically significant sex differences that remained were associated with effect sizes that are considered small, thus demonstrating generalizability of the MNS regression norms to a different geographic region of the United States.

A similar pattern was present when examining AVLT scores across age, with the MNS demographic adjustment yielding demographically corrected *T* scores near the idealized value of *T* = 50 with small effect sizes that did not differ statistically across groups. The robustness of the demographic MNS normative regression equations in adjusting for demographic differences observed with raw scores provides strong support for their clinical application. It is noteworthy that although our EHBS sample includes participants with relatively high educational levels, the average primary AVLT *T* scores remain close to *T* = 50 reflecting appropriate MNS demographic adjustment. The utility of demographically corrected MNS scores has been demonstrated in improved amnestic mild cognitive impairment (aMCI) identification, with failure to make appropriate sex-based performance correction leading to aMCI diagnosis associated with a 20% diagnostic error rate (Sundermann et al., 2019).

### Confidence interval application

CIs associated with an obtained score help minimize clinical judgment errors that may arise from over-interpretation of chance fluctuations, although CIs are often neglected in test score interpretation. CIs help determine whether an observed score is different from a population parameter (e.g., 1.5 standard deviations below the mean criterion for suspected cognitive impairment), or used to test whether a score at retest clearly falls above or below the score obtained at a prior assessment. Innovative approaches to establishing CIs have relied on bootstrapping approaches from large datasets (i.e., 10,000+) to estimate percentile precision at lower percentile levels (O’Connell et al., 2021). This approach demonstrated the superiority of different age-based regression models for predicting 5th percentile performance based on sex and education level as characterized by measurement invariance of different models, but revealing variability in the methods employed to adjust for demographic covariates. This hybrid approach to normative performance generation at specific percentile thresholds has an advantage since it is specifically designed to minimize measurement bias at cut scores commonly used to infer abnormal cognitive ability. However, one limitation of the approach described by O’Connell and colleagues is that it does not incorporate retest reliability estimates, so may consequently underestimate regression to the mean effects. Further comparisons of alternative approaches are needed.

Although test-retest reliabilities are typically included in formal testing manuals, they often are calculated from short time intervals (e.g., CVLT-II retest interval *Mdn* = 21 days, *R* = 9–41; Delis et al., 2000). Further, except for global measures of cognitive abilities (e.g., WAIS-IV), reliabilities are typically not incorporated into CIs despite their importance for valid test inferences (Bowden & Finch, 2017; Franzen, 2000; Nunnally & Bernstein, 1994). CIs derived from MNS test-retest reliability coefficients are particularly valuable since they reflect relatively long follow-up intervals (*M* = 16.7 months), minimizing carry over learning/memory effects from using the same stimuli that inflate test-retest reliability estimates.

The appropriate midpoint anchor for CIs is not the *observed score*, but rather the *predicted true score*. The *predicted true score* reflects the influence of regression to the mean upon retest, when the retest score is likely to be closer to the population mean. Thus, the *predicted true score* will always fall between the observed score and the population mean (Bowden & Finch, 2017; Nunnally & Bernstein, 1994). The value of the *predicted true score* is determined by the score reliability using the following formula: *predicted true score* = (observed score * reliability) + ([population mean * (1-reliability]). Thus, with an approximate reliability of .8 for Trial 1–5 Sum (Table 8), an *observed T* score of 40 will be associated with a *predicted true T score* of 42 (i.e., [40 * .8] + [50 * .2], or [32 + 10]). Rather than reporting the score and 90% CI as *T* = 40 (90% CI 33–47), the appropriate band of uncertainty around the score is more accurately reported as *T* = 40 (90% CI 35–49). Table 7 contains predicted true scores and associated CIs for a range of AVLT Trial 1–5 *T* values often used to infer atypically low AVLT learning performance. Lower reliabilities result in bigger adjustments from observed score to *predicted true score* (see Bowden & Finch, 2017).

The classification of amnestic Mild Cognitive Impairment (aMCI) (or mild neurocognitive disorder) is often based upon memory performance that is at least 1.5 *SD* below the population mean. Consequently, scores that are within 1.5 *SD* of the mean, but which are associated with a CI that includes the −1.5 *SD/T* = 35 threshold may not be interpreted as excluding aMCI. Conversely, an observed score that is below the −1.5 *SD/T* = 35 can only be interpreted as indicating aMCI with 90% confidence if the associated 90% CI does not include the −1.5 *SD/T* = 35 threshold score. As seen in Table 8, a *T* = 40 which is typically interpreted as reflecting low average performance (16th percentile) includes the −1.5 *SD/T* = 35 threshold in its CI and is consistent with aMCI given an appropriate clinical context and supporting history suggesting memory decline. Alternatively, a score of *T* = 30 corresponding to a 2nd percentile performance contains *T* scores up to *T* = 41 in its CI, demonstrating that a score this low on AVLT may be consistent with normal ability. Failure to use CIs that use the predicted true score as the appropriate midpoint for the CI will increase the risk of diagnostic error since scores needed to infer impairment occur at the lower end of the distribution in which regression to the mean associated with performance improvement upon retesting is more likely than obtaining a lower score (see Bowden & Finch, 2017).

### Recognition

Examination of AVLT recognition provides information to potentially guide future test modifications. The most frequent incorrect item selected was *face*, and although participants are instructed to identify only items from the AVLT word list, *face* is one of the five memory items from the MoCA. The frequency of choosing this foil is disproportionately high reflecting source memory confusion and would not be expected when the AVLT is administered without prior MoCA stimulus exposure. Although we have not altered the EHBS assessment protocol, we have changed *face* on our AVLT recognition form to *finger* for our clinical use because the MoCA is included in our telehealth assessment protocols (Hewitt & Loring, 2020). The next most common foils selected as a target are *teacher* (22.4%) and *gun* (12.3%). We speculate that the high frequency of teacher identification is related to the presence of school as a target item. The high frequency of *gun* selection relates not only to source memory confusion since it is a List B word, but there may be additional influences of it being an emotionally charged item that may contribute to its attractiveness as a distractor, and which may be expected to have different saliency in different cultures or environments. There are multiple recognition word lists provided by Schmidt (1996) from which to choose, many of which explicitly test recognition for both List A and List B words with versions that also contain foils that are semantically related to the List A targets.

There were two recognition foils never identified as targets – *kerchief* and *broomstick*. These words appear antiquated, are not part of the contemporary vernacular in North America, and likely are not viewed as attractive distractors since they are colloquially distinct from other targets. While *kerchief* may be more common in other cultures, similar words such as *handkerchief* or *bandana* may be better foils for recognition testing, realizing that both target words and distractors will likely vary in their selection/saliency based upon cultural influences. We are not aware of any rules of thumb to create foil items for recognition memory testing, but as an initial approach, the likelihood of foil selection in cognitively healthy participants should probably be modest (e.g., ≤ 5%).

Characterization of the performance of individual items is a novel aspect of this report, although future studies should benefit from applying more advanced approaches including differential item functioning. Few neuropsychological measures have been subjected to measurement invariance or differential item function analyses; however, the presence of differential item functioning within a given scale can result in different clinically relevant thresholds across groups. For example, both sociodemographic factors and primary language have been demonstrated to exert strong effects of task performance (e.g., Jones, 2006; Yang et al., 2009) such that geographic region and education may be most relevant to characterize with differential item functioning.

The primary limitation of this report is its restricted range for both age and education. The MNS sample included subjects ranging from 30–91 years with education levels ranging from 8–20 years (Stricker et al., 2021). The number of MNS participants with less than a high school education cannot be determined, although the MNS sample included 28.4% with education categorized as between 8–12 years while there were no subjects in this validation sample who had not completed high school at a minimum. Thus, this report does not provide empirical evidence to support MNS application outside of these ranges. While a linear relationship with memory change for younger ages than those included in this report can be expected in a healthy population, it is more likely that there is a nonlinear relationship between education and memory performance at lower education levels (Lövdén et al., 2020) and application of normative MNS to subjects with low education should be interpreted with appropriate caution.

Despite shortcomings of being a nonproprietary verbal memory measure without formal standardization, many of which are addressed by the MNS normative project, the AVLT remains a popular test of verbal learning and memory. For example, the AVLT was selected as a Common Data Element for verbal memory assessment by the National Institute of Neurological Disorders and Stroke for funded epilepsy studies due to its greater sensitivity than the CVLT to verbal memory impairment associated with left temporal lobe seizure onset (Loring et al., 2011). This increased sensitivity was hypothesized to be related to the AVLT’s use of semantically unrelated words. Since the CVLT stimulus items are semantically related, patients may use this relationship for self-cueing during recall, thereby partially compensating for disease related memory inefficiencies (Loring et al., 2008). The AVLT is also a common memory measure for longitudinal research studies in aging and dementia such as the *Alzheimer’s Disease Neuroimaging Initiative* (Mueller et al., 2005) and the *Advance Cognitive Training for Independent and Vital Elderly* (Tennstedt & Unverzagt, 2013). The AVLT’s popularity is also demonstrated by its modification for use in multiple languages including Spanish (Ponton et al., 1996), Portuguese (Malloy-Diniz et al., 2007), German (Helmstaedter et al., 2001), Czech (Bezdicek et al., 2014), Russian (Melikyan et al., 2020) as well as Rey’s original French word list (Sziklas & Jones-Gotman, 2008) to name but a few.

This is the first study we are aware of to characterize performance improvement associated with recognition testing compared to delayed free recall in a cognitively healthy cohort. In clinical practice, a large performance benefit is often interpreted as evidence of retrieval inefficiency, although this series demonstrates that the recognition benefit after age 65 averages 5 words or more. This age effect is not surprising, but demonstrates that relatively large recognition performance benefit is common in normal aging and does not, by itself, suggest the presence of disease-related retrieval inefficiency in similarly aged patients (e.g., retrieval deficit hypothesis with Parkinson disease, see Flowers et al., 1984; but also see Whittington et al., 2000).

In conclusion, this report confirms a strong sex effect across multiple AVLT measures in addition to age and education, but also demonstrates the overall accuracy of MNS normative data to correct for these demographic differences, at least for the age and education ranges examined. Further support for MNS use in performance characterization is present by its ability to adjust age-related performance differences to overall performance levels near the expected value of *T* = 50. It is a testament to both Claparède’s and Rey’s thoughtfulness in developing a technique to measure memory that the AVLT remains an important verbal memory test in the 21st century.

## Figures and Tables

**Table 1.** Raw performance levels and demographically corrected MNS *T* scores across age groups. Standard deviations for both scores are In parentheses

Raw scores	50–54 (*n* = 93)	55–69 (*n* = 128)	60–64 (*n* = 170)	65–69 (*n* = 163)	70–74 (*n* = 104)	75–79 (*n* = 5)	*η* ^*2*^
Trial 1–5 sum	52.5 (8.6)	51.0 (8.9)	47.9 (9.1)	46.3 (9.5)	45.4 (9.6)	40.0 (10.7)	.079
3-Trial sum	27.6 (5.7)	26.5 (5.6)	24.7 (5.6)	23.9 (5.5)	23.6 (5.5)	21.2 (5.8)	.066
List B	6.4 (2.1)	6.1 (1.8)	6.0 (1.7)	5.7 (1.7)	5.5 (1.8)	5.4 (1.5)	.025
Immediate recall	11.1 (2.9)	10.5 (3.3)	9.3 (3.6)	9.1 (3.5)	8.7 (3.4)	7.0 (4.2)	.065
Delayed recall	11.2 (2.9)	10.5 (3.3)	9.3 (3.6)	8.7 (4.0)	8.3 (3.6)	7.4 (4.6)	.077
Recognition hits	13.9 (1.4)	13.6 (1.7)	13.5 (2.0)	13.3 (2.0)	12.8 (2.8)	13.4 (1.6)	.025
Recognition false positives	0.9 (0.8)	0.9 (0.9)	1.0 (1.0)	1.1 (1.0)	1.0 (1.0)	0.6 (1.0)	.006
Recognition-discrimination	13.1 (1.7)	12.7 (2.0)	12.5 (2.3)	12.2 (2.6)	11.8 (2.9)	12.8 (2.0)	.026
MNS *T* scores	50–54	55–69	60–64	65–69	70–74	75–79	*η* ^*2*^
Trial 1–5 sum	51.5 (10.9)	51.2 (11.0)	49.8 (10.8)	50.2 (11.6)	51.9 (12.0)	49.2 (11.4)	.005
3-Trial sum	51.9 (11.8)	51.4 (11.8)	49.7 (11.4)	50.3 (12.2)	52.4 (12.0)	52.4 (12.4)	.007
List B	52.9 (11.0)	53.1 (10.6)	54.5 (10.5)	54.4 (11.4)	55.0 (11.4)	57.6 (9.9)	.006
Immediate recall	50.7 (10.3)	50.1 (11.3)	48.6 (10.6)	49.4 (12.6)	49.8 (11.6)	47.2 (13.3)	.004
Delayed recall	52.5 (10.2)	52.1 (10.9)	50.4 (11.4)	50.2 (13.0)	51.2 (11.4)	52.6 (13.7)	.006

*Notes*: All group differences statistically significant the *p* < .001 level or better with exception of List B and Recognition Hits (both *p* = .006), False Positives (*p* = .009), Recognition false Positives (NS), and Recognition/Discrimination (*p* = .004).

By convention, *η*^2^ ≥ .01 is considered a small effect, *η*^2^ ≥ .06 is considered a medium effect, and *η*^2^ ≥ .14 is considered a large effect.

**Table 2.** Raw performance levels and demographically corrected MNS *T* scores for females and males. Standard deviations for both scores are In parentheses

Raw scores	Sample	Female	Male	Total	*η* ^*2*^
Trial 1–5 sum	*n* = 663	50.0 (9.0)	44.4 (9.5)	48.4 (9.5)	.074
3-Trial sum	*n* = 663	26.0 (5.6)	22.3 (5.5)	25.0 (5.7)	.059
List B	*n* = 660	6.0 (1.8)	5.5 (1.8)	5.9 (1.9)	.018
Immediate recall	*n* = 659	10.1 (3.2)	8.6 (3.8)	9.6 (3.3)	.038
Delayed recall	*n* = 646	10.1 (3.5)	8.1 (3.8)	9.5 (3.7)	.060
Recognition hits	*n* = 660	13.7 (1.8)	12.8 (2.3)	13.4 (2.0)	.045
Recognition false positives	*n* = 660	0.9 (1.0)	1.0 (1.0)	1.0 (1.0)	.010
Recognition-discrimination	*n* = 660	2.8 (2.1)	11.6 (2.5)	12.4 (2.3)	.053
MNS *T* scores	Sample	Female	Male	Total	*η* ^*2*^
Trial 1–5 sum	*n* = 663	50.1 (11.0)	52.1 (11.6)	50.7 (11.2)	.007
3-Trial sum	*n* = 663	50.6 (11.6)	51.8 (12.3)	50.9 (11.8)	.002
List B	*n* = 660	53.7 (10.6)	55.0 (11.1)	54.1 (10.7)	.003
Immediate recall	*n* = 659	49.0 (11.2)	50.9 (11.5)	49.6 (11.4)	.006
Delayed recall	*n* = 646	49.7 (11.5)	51.0 (12.1)	50.1 (11.7)	.002

*Notes*: All group differences with raw scores are statistically significant at the *p* < .001 level or better with exception of False Positives, which is statistically significant at *p* = .009.

By convention, *η*^2^ ≥ .01 is considered a small effect, *η*^2^ ≥ .06 is considered a medium effect, and *η*^2^ ≥ .14 is considered a large effect.

**Table 3.** Raw performance levels and demographically corrected MNS *T* scores across education groups. Standard deviations for both scores are in parentheses

Raw scores	12 years (*n* = 11)	13–15 years (*n* = 98)	16–17 years (*n* = 274)	18+ years (*n* = 280)	*η* ^*2*^
Trial 1–5 sum	44.6 (7.6)	46.0 (9.9)	48.0 (9.1)	49.6 (9.7)	.020
3-Trial sum	21.7 (4.0)	24.1 (6.1)	24.8 (5.4)	25.8 (5.9)	.017
List B	5.7 (1.9)	5.3 (1.7)	5.9 (1.9)	6.1 (1.8)	.020
Immediate recall	9.3 (3.9)	9.0 (3.3)	9.6 (3.4)	9.9 (3.2)	.009
Delayed recall	9.0 (4.4)	9.1 (3.5)	9.5 (3.6)	9.7 (3.8)	.003
Recognition hits	13.2 (2.4)	13.2 (2.5)	13.5 (1.9)	13.4 (2.0)	.004
Recognition false positives	1.3 (1.3)	1.2 (1.0)	1.0 (1.0)	0.9 (1.0)	.012
Recognition-discrimination	11.9 (2.4)	12.0 (2.7)	12.5 (2.1)	12.5 (2.3)	.008
MNS *T* scores	12 years	13–15 years	16–17 years	18+ years	*η* ^*2*^
Trial 1–5 sum	50.4 (6.8)	50.3 (12.5)	50.5 (10.7)	51.1 (11.4)	.001
3-Trial sum	48.0 (7.7)	51.3 (13.3)	50.4 (11.2)	51.4 (12.0)	.003
List B	56.8 (12.4)	52.6 (10.2)	54.4 (10.8)	54.2 (10.8)	.004
Immediate recall	50.9 (12.7)	48.9 (11.9)	49.6 (11.8)	49.7 (10.8)	.001
Delayed recall	51.1 (13.3)	50.0 (11.8)	50.4 (11.6)	49.8 (11.7)	.001

*Notes*: Significant group differences were present for Trial 1–5 Sum (*p* = .004), 3-Trial Sum (*p* = .009), List B (*p* = .004), and False Positive Recognition Flits (*p* = .045).

By convention, *η*^2^ ≥ .01 is considered a small effect, *η*^2^ ≥ .06 is considered a medium effect, and *η*^2^ ≥ .14 is considered a large effect.

**Table 4.** Item-level effect sizes for age, sex, and education differences for Individual AVLT stimulus words

	*η*^*2*^ (age)	*η*^*2*^ (sex)	*η*^*2*^ (education)
Drum	.020	.017	.002
Curtain	.020	.008	.008
Bell	.032	.012	.008
Coffee	.036	.025	.014
School	.021	.030	.003
Parent	.030	.032	.007
Moon	.037	.040	.007
Garden	.029	.060	.010
Hat	.011	.013	.007
Farmer	.022	.005	.011
Nose	.024	.016	.010
Turkey	.029	.007	.013
Color	.041	.017	.013
House	.017	.000	.015
River	.006	.000	.004

*Notes*: By convention, *η*^2^ ≥ .01 is considered a small effect, *η*^2^ ≥ .06 is considered a medium effect, and *η*^2^ ≥ .14 is considered a large effect.

**Table 5.** Item-level recognition identification for targets and foils (F = 463, M = 200)

Targets	Identified (F, M)	Foils	Identified (F, M)
Coffee	96.1% (97.4%, 93.0%)	Face	46.2% (44.1%, 51.0%)
Farmer	95.8% (96.3%, 94.5%)	Teacher	22.4% (20.2%, 27.5%)
Curtain	94.2% (95.2%, 92.0%)	Gun	12.3% (11.5%, 14.0%)
Parent	93.6% (95.0%, 90.5%)	Bridge	3.8% (3.5%, 4.5%)
Moon	92.9% (95.7%, 86.5%)	Pen	3.5% (3.5%, 3.5%)
Turkey	92.9% (95.4%, 87.0%)	Road	3.3% (2.8%, 4.5%)
Garden	92.7% (95.0%, 87.5%)	Floor	2.1% (1.5%, 3.5%)
Drum	92.1% (93.5%, 89.0%)	Classroom	1.7% (1.3%, 2.5%)
School	91.4% (92.0%, 90.0%)	Soldier	1.1% (1.5%, 0.0%)
Bell	87.1% (89.8%, 81.0%)	Beet	0.8% (0.7%, 1.0%)
River	86.4% (87.6%, 83.5%)	Minute	0.6% (0.4%, 1.0%)
Nose	85.2% (88.0%, 78.5%)	Children	0.5% (0.4%, 0.5%)
Hat	83.0% (87.0%, 74.0%)	Forehead	0.2% (0.2%, 0.0%)
Color	80.3% (83.0%, 74.0%)	Kerchief	0.0% (0.0%, 0.0%)
House	78.2% (79.1%. 76.0%)	Broomstick	0.0% (0.0%. 0.0%)

**Table 6.** Recognition benefit (standard deviation) across age groups

	50–54	55–69	60–64	65–69	70–74	75–79	*η* ^*2*^
Raw scores	2.7 (2.3)	3.1 (2.7)	4.1 (3.0)	4.6 (3.3)	4.7 (2.7)	6.0 (3.7)	0.066

Notes: By convention, *η*^2^ ≥ .01 is considered a small effect, *η*^2^ ≥ .06 is considered a medium effect, and *η*^2^ ≥ .14 is considered a large effect.

**Table 7.** Confidence intervals estimated for primary AVLT scores using both the *SE*_*Estimation*_ and *SE*_*Prediction*_ for raw scores and *T* scores, respectively. Note that confidence intervals should be centered on the predicted true score (see text for details)

Measure		Pearson coefficient^a^	*SD* ^b^	*SE*_Estimation_	Single score 90% CI	*SE*_Prediction_	Interval change 90% CI
Trial 1–5 sum	Raw	.798	10.0	4.01	13.2	6.03	19.82
	*T* score	–	10	4.01	13.2	6.03	19.82
3-Trial sum	Raw	.732	5.5	2.44	8.02	3.75	12.32
	*T* score	–	10	4.43	14.58	6.81	22.42
List B	Raw	.507	1.7	0.85	2.80	1.47	4.81
	*T* score	–	10	5.00	16.44	8.62	28.36
Immediate recall	Raw	.737	3.3	1.45	4.78	2.23	7.34
	*T* score	–	10	4.40	14.48	6.76	22.24
Delayed recall	Raw	.761	3.5	1.49	4.92	2.27	7.46
	*T* score	–	10	4.26	14.02	6.49	21.34

a MNS test-retest Pearson reliabilities (Stricker et al., 2021, Table 3).

b MNS raw score Standard Deviations (Stricker et al., 2021, Supplemental Table 1).

**Table 8.** Confidence intervals estimated for 4 AVLT *T* score thresholds representing for trial 1–5 sum. Note that for obtained *T* = 25, the lower CI limit does not practically extend lower than *T* = 20

	Predicted true score	Single score 90% CI	Score and CI
*T* = 40	*T* = 42	CI = 13.2	*T* = 40 (90% CI 34.9–49.1)
*T* = 35	*T* = 38	CI = 13.2	*T* = 35 (90% CI 30.9–45.1)
*T* = 30	*T* = 34	CI = 13.2	*T* = 30 (90% CI 26.9–41.1)
*T* = 25	*T* = 30	CI = 13.2	*T* = 25 (90% CI 20.0–37.1)
